# Diagnosis difficulties in a patient with progressive loss of vision - a case report

**DOI:** 10.22336/rjo.2017.11

**Published:** 2017

**Authors:** Teodor Razvan Cristescu

**Affiliations:** * Ploiesti County Hospital, Ploiesti, Romania; Miroptic Med, Ploiesti, Romania

**Keywords:** cancer-associated retinopathy, paraneoplastic retinopathy, autoimmune retinopathy

## Abstract

The paper presents the case of a 57-year-old male patient who complained of slow progressive loss of visual acuity. Anamnesis revealed he was a heavy drinker and he was previously diagnosed with a pancreatic cancer, observed on the MRI. The clinical examination revealed ocular features that made the diagnosis difficult. Initially, it seemed to be a case of narrow angle glaucoma but further ocular examinations revealed macular thinning.

## Introduction

**Case report**

**Fig. 1:**
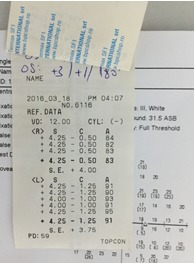
Refraction

A 57-year-old man presented to the ophthalmology department complaining of progressive visual loss, which he had been suffering from for the last few weeks, wishing to undertake surgery for cataract. Anamnesis revealed he was a heavy drinker and that he was recently discovered with pancreatic cancer (observed on the MRI). He was not taking any medication but he mentioned he was scheduled for a consultation with the oncologist. At presentation, his visual acuity was 1/ 10 with his own corrective glasses at both eyes. Measured refraction was around +4 spherical diopters (**Fig. 1**) but the maximal correction did not provide a better visual acuity. Intraocular pressure measured by a non-contact tonometer was 9 in his right eye and 14 in his left eye.

**Fig. 2:**
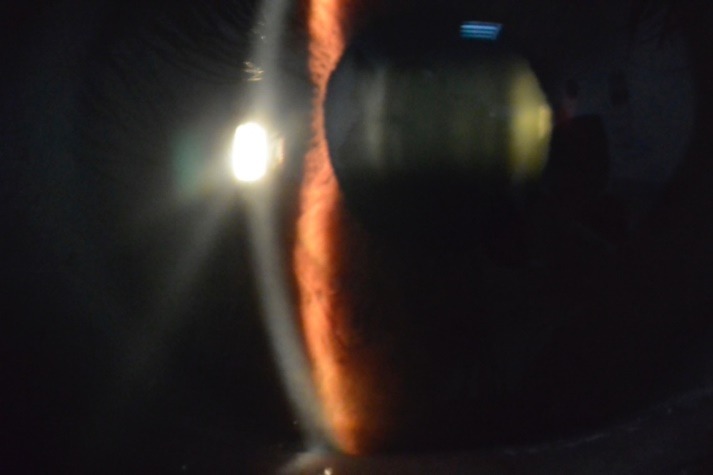
Anterior aspect

**Fig. 3:**
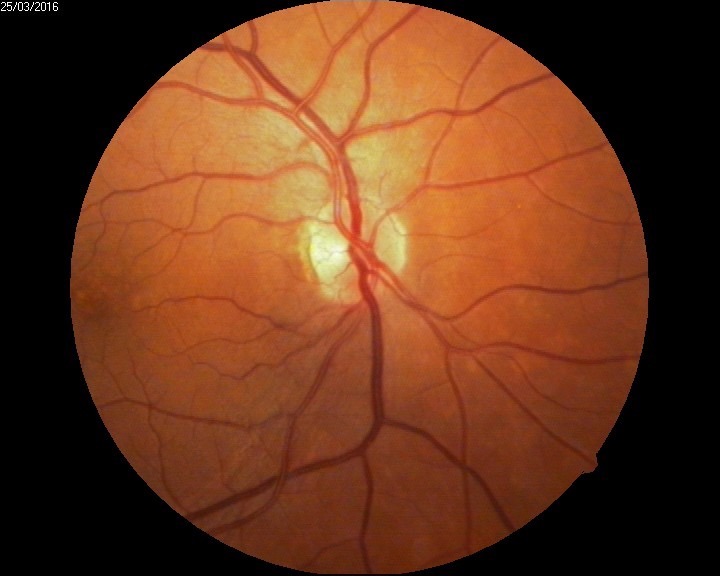
Fundus examination RE

**Fig. 4:**
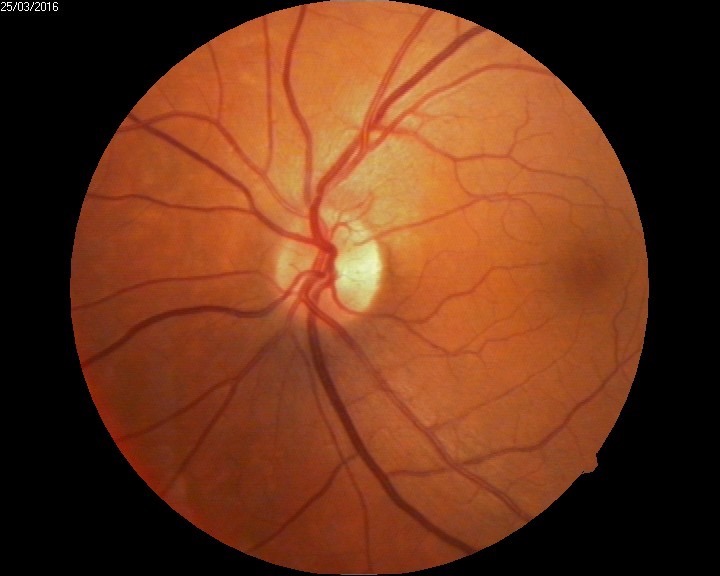
Fundus examination LE

**Fig. 5:**
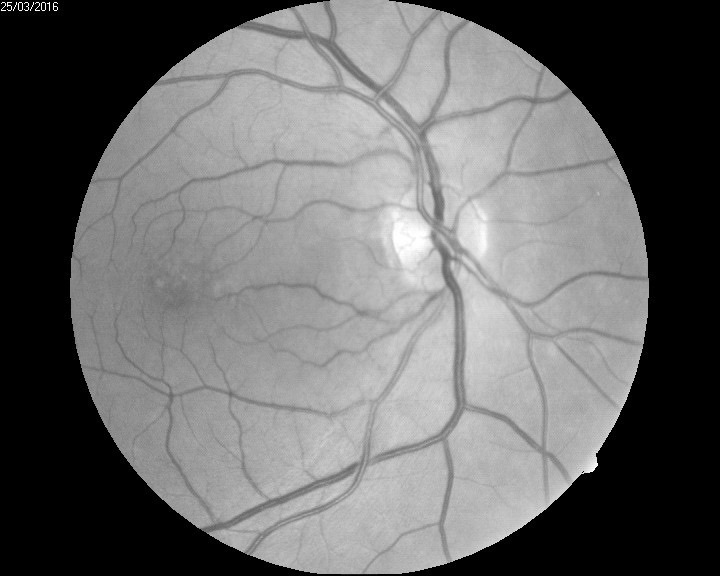
Red free image REF

**Fig. 6:**
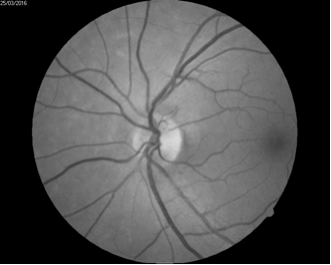
Red free image LEF

The biomicroscopic examination revealed a shallow anterior chamber and a clear lens (**Fig. 2**). Due to this anatomical conformation, narrow angle glaucoma was suspected but the patient did not recall any episodes of ocular pain, redness, and acute loss of visual acuity. The fundus examination was performed under a careful dilatation, with tropicamide and revealed a few soft macular drusen in the right eye, and a curious sectorial temporal pallor of both optic nerve heads (**Fig. 3**,**4**). Red free images of the posterior pole were taken but did not reveal any loss of ganglionar nerve fibers suggestive of glaucoma (**Fig. 5**,**6**).

**Fig. 7:**
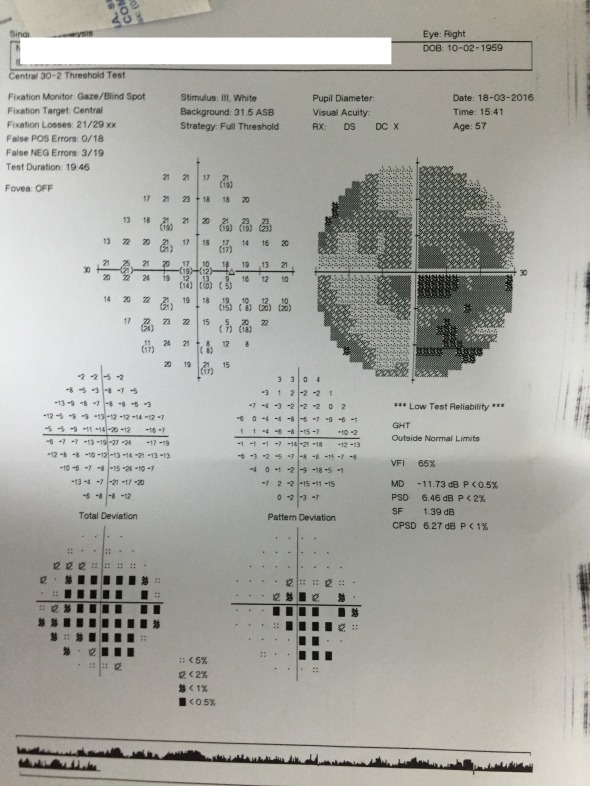
Visual field RE

**Fig. 8:**
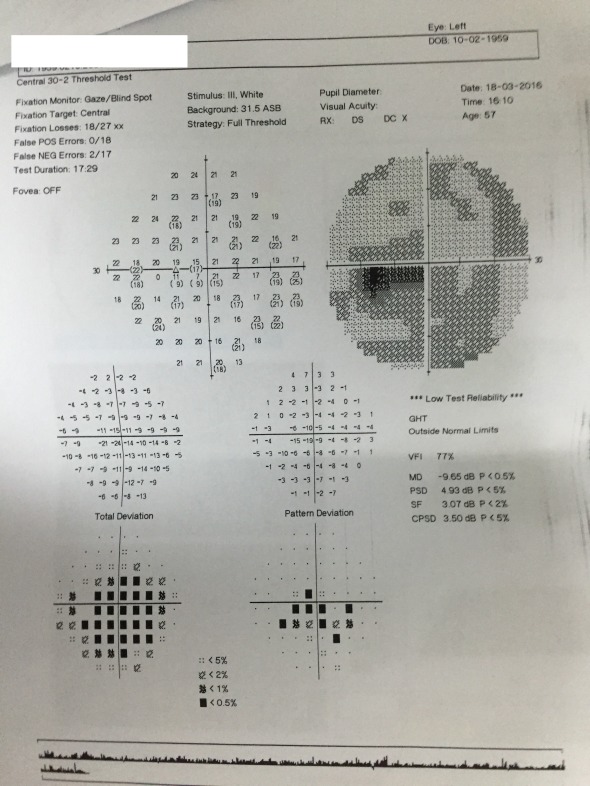
Visual field LE

Humphrey visual field examination revealed central scotomas in both eyes. The patient was unable to fixate, hence a lot of fixation errors (**Fig. 7**,**8**) appeared. These results, which were not specific for glaucoma, oriented more towards a macular disease or some kind of optic nerve disease (neuritis or AION). Optical coherence tomography for both maculae and optic nerve heads was recommended for further investigation.

**Fig. 9:**
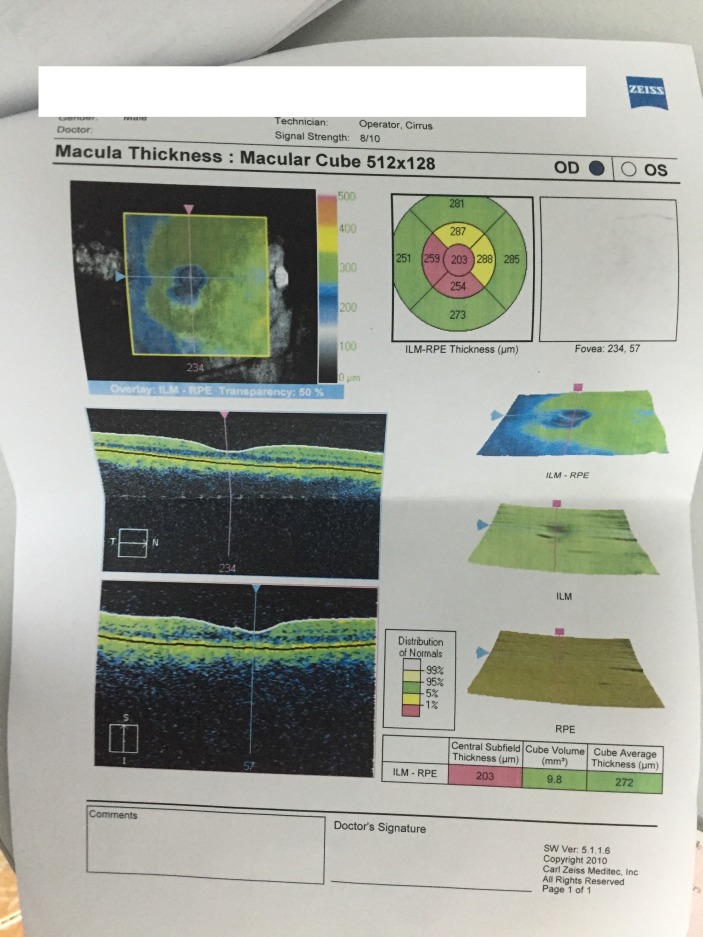
OCT LE

**Fig. 10:**
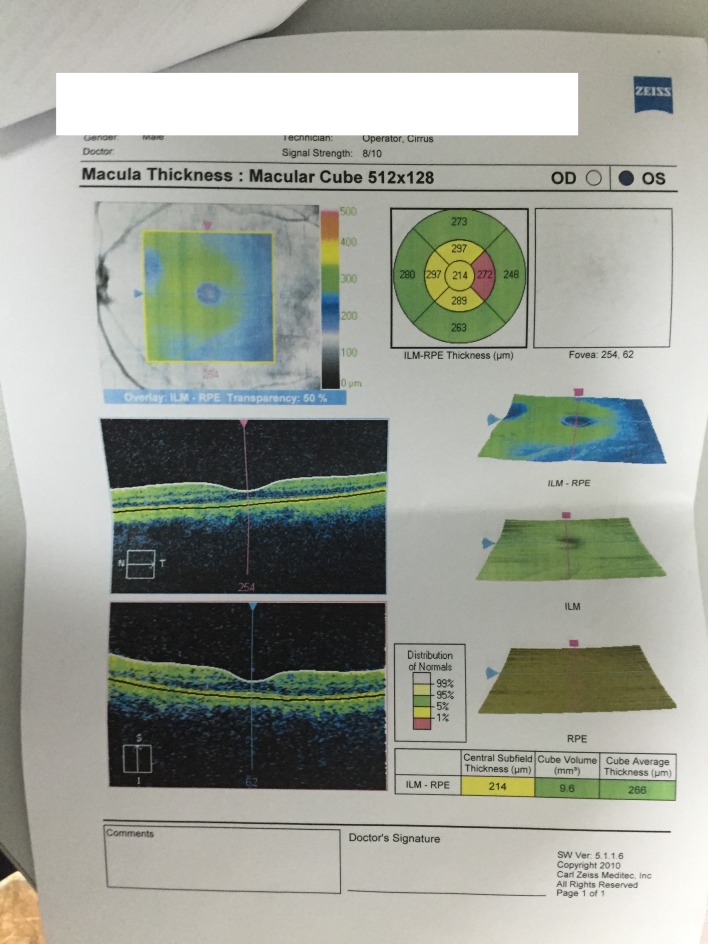
OCT RE

Optical coherence tomography revealed a macula thinner than average, highlighted by the red color on the thickness map (**Fig. 9**,**10**). OCT of the optic nerve head and RNFL analysis showed a thick neuroretinal rim, with no evidence of optic nerve atrophy. The results suggested a macular disease. 

**Fig. 11:**
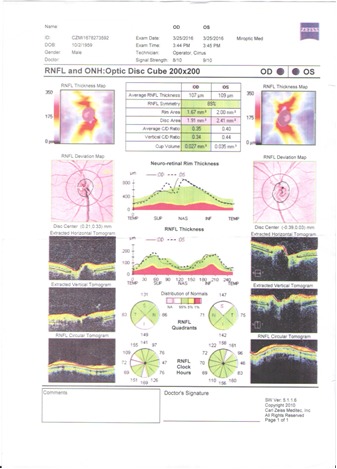
OCT optic nerve

## Diagnosis

The clinical signs and paraclinical examinations alone were not sufficient for a positive diagnosis, but as the patient revealed documents about his general health, mentioning that he was diagnosed with pancreatic cancer, the diagnosis of a cancer associated retinopathy (CAR) seemed plausible. No diagnostic criteria were set for this type of paraneoplastic retinopathy. Apart from the above mentioned criteria, one of the most important was the identification of serum anti-retinal antibodies, the most important of these being the anti-recoverin (a 23-kDa protein) and the anti-alpha-enolase antibody (46-kDa) [**6**,**7**]. USA has tests for the identification of these antibodies. On the other hand, these antibodies are not diagnostic tools for CAR themselves because they were also identified in normal individuals, so they must be correlated with the clinical findings.

## Differential diagnosis

1. subacute or intermittent angle closure glaucoma - visual field defects were not characteristic for glaucomatous damage. The optic nerve OCT revealed a normal width of the neuroretinal rim.

2. toxic neuropathy - the patient was a heavy drinker

3. retrobulbar neuritis or ischemic neuropathy - the paraclinical investigations localized the disease at the level of the macula and not at the level of the optic nerve.

4. retinal dystrophies

5. optic nerve tumors - The MRI revealed only the pancreatic mass, with no sign of other affected organs.

## Treatment

The patient followed the oncologist for the treatment of his cancer. At this moment, there is no known effective treatment for CAR. The treatment of systemic cancer does not lead to the improvement of vision. Many systemic immunosuppressive medications have been tried with mild and transient improvement in the visual acuity but no long lasting improvement has been recorded [**1**].

## Evolution and prognostic

The patient could not be followed-up for more than a few weeks because he did not come to the ophthalmological evaluations anymore. The prognosis for visual recovery in CAR was poor, but, on the other hand, complete or further progressive visual loss did not occur. Usually, disease stabilization occurs in such a case.

## Case particularities

Although initially the patient seemed to have a form of angle closure glaucoma due to his shallow anterior chamber, further exams showed a thinned macula and no objective signs of nerve damage.

Cancer associated retinopathy is a rare disease that is most often associated with colon or gynecologic cancers. The presented case associated a maculopathy and a pancreatic cancer.

## Discussions

The presented case closely resembled 2 other cases that were recently presented by Eadie et al. They reported the cases of 2 women with localized foveal flattening and history of cancer. The OCT findings showed the flattening of the foveal depression in both eyes with the disruption of the inner retinal layers [**2**].

Cancer associated retinopathy is a specific type of paraneoplastic disease of the eye, associated with the presence of extraocular malignancy and circulating autoantibodies against retinal proteins. It is hypothesized that some tumors express protein antigens that are the same or cross-react with retinal proteins [**3**].

CAR is triggered by an alteration of the immune system. The autoimmune reaction leads to retinal photoreceptor cell death. In patients with a diagnosis of systemic cancer, screening should be done for anti-retinal antibodies, in particular anti-recoverin (23-kDa protein) [**4**] and anti-alpha-enolase (46-kDa) [**5**].

The first retinal antigen shown to represent the source of autoimmunity in CAR was a 23 kDa protein named recoverin [**6**], but many other proteins were found to be antigenic (alfa-enolase [**7**], transducin).

Electroretinography (ERG) is very important to highlight the retinal dysfunction. Full field ERG is abnormal in most cases (attenuated or absent photopic and scotopic response) [**8**]. In cases in which only the cones are affected, full field ERG may be normal but multifocal ERG reveals the macular disease. OCT is helpful in evaluating patients with CAR and the studies published so far showed reduced central macular and foveal thickness [**9**].
